# Chemosensory dysfunction, Oral disorders and Oral health-related quality of life in patients with primary Sjögren’s syndrome: comparative cross-sectional study

**DOI:** 10.1186/s12903-020-01169-5

**Published:** 2020-07-03

**Authors:** Mirjana Šijan Gobeljić, Vera Milić, Nada Pejnović, Nemanja Damjanov

**Affiliations:** 1 Institute of Rheumatology, Resavska 69, Belgrade, 11000 Serbia; 2 University of Belgrade Medical School, Institute of Rheumatology, Belgrade, Serbia

**Keywords:** Sjögren’s syndrome, Saliva secretion, Oral disorders, Chemosensory dysfunction, Quality of life

## Abstract

**Background:**

The aim of this study was to evaluate chemosensory function and oral disorders in patients with primary Sjögren’s syndrome (pSS) and to compare these findings with those of age- and gender-matched healthy controls.

**Methods:**

This comparative cross-sectional study included 58 patients with primary Sjögren’s syndrome (pSS) and 55 age- and gender-matched healthy controls. Olfactory and gustatory function, burning sensations in the tongue (BST) and halitosis were assessed. Oral health-related quality of life (OHRQoL) was evaluated using the short-form Oral Health Impact Profile (OHIP-14).

**Results:**

Patients with pSS had significantly lower self-reported visual analogue scale (VAS) smell score (8.6 ± 2.2 vs. 9.6 ± 0.7, *p* = 0.016) and VAS taste score (8.5 ± 2.1 vs. 9.5 ± 0.7, *p* = 0.014) than healthy controls. A greater proportion of patients with pSS had anosmia (3.8% vs. 0.0%) or hyposmia (36.5% vs. 13.2%) and ageusia for basic tastes: sweetness (34.0% vs. 7.5%), sourness (10.6% vs. 0.0), saltiness (10.0% vs. 5.7%) or bitterness (19.1% vs. 1.9%) as evaluated using Sniffin Sticks test and taste stripts, respectively. A higher proportion of pSS patients complained of dysgeusia (52.6% vs. 9.4%, *p* < 0.0001) and BST (45.6% vs. 0.0%, p < 0.0001), while similar number of patients with pSS and controls reported halitosis (31.6% vs. 28.3%, *p* = 0.434). The mean OHIP-14 score was significantly higher in patients with pSS (6.8 ± 7.0 vs. 2.3 ± 8.5, *p* < 0.001) indicating patients’ poorer OHRQoL compared with controls.

**Conclusions:**

The majority of patients with pSS had impaired chemosensory function and indicators of oral health in comparison with the age- and gender-matched healthy controls. Further studies of oral hygiene habits and dietary intake of these patients are needed to ensure better management of oral health problems in patients with pSS.

## Background

Sjögren’s syndrome (SS) is a chronic, systemic, autoimmune disease with the prevalence between 0.05 and 1% in European population. Exocrine glands, especially salivary and lacrimal glands, are mainly affected leading to dryness of the mouth and/or eyes. Fatigue, joint and muscle pain are commonly present in patients with SS. Sjögren’s syndrome can be further subclassified into primary disease (primary Sjögren syndrome, pSS) and secondary disease (secondary Sjögren syndrome, sSS), when it is associated with another connective tissue disease [1].

SS is triggered in genetically predisposed individuals by environmental factors, such as infectious agents. The complex pathogenesis of SS is characterized by dysfunction of innate and adaptive immunity. . The hallmarks of SS are lymphocytic infiltration of the exocrine glands and the presence of circulating autoantibodies (anti-Ro/SS-A, anti-La/SS-B) [2], as well as autoantibodies directed against muscarinic acetylcholine type 3 receptors (M3R), which functionally inhibit salivary secretion [3].

There is no cure for SS. Patients with SS receive mainly symptomatic treatment, which is why artificial tears and saliva are recommended as standard substitution therapy. Dental erosions, dental caries, mucosal infection, ulcers and oral candidiasis are commonly present in patients with SS; they are related to a decrease in salivary flow and the qualitative changes in saliva. Due to oral cavity dryness (xerostomia), chewing, swallowing, speech and sleep may be affected, resulting in impaired quality of life in patients with pSS [4].

Chemosensory disorders have been reported in patients with pSS [5, 6]. Chemosensory disorders, which include olfactory and gustatory dysfunction, could manifest as reduced ability, distortion or absence of the senses of taste and/or smell [7, 8]. Many patients with chemosensory disorders experience a burning sensations or numbness in the mouth, especially in or on the tongue, the sensations that may originate in the gustatory nerve fibers [9]. Burning mouth syndrome (BMS) is defined as a burning sensation in the tongue (BST), or burning in some other mucosal membranes, which lasts for at least 4–6 months [10]. Patients with SS and patients with burning mouth syndrome (BMS) usually have similar oral complaints; however, these diseases have different etiology, pathogeneses, diagnostic criteria, and treatment [11]. Halitosis, (oral mal-odor, defined as an unpleasant breath odor of oral or extra-oral origin), is another common oral complaint that can be associated with low salivary secretion or chemosensory disorders in patients with pSS [12]. Dysgeusia, BST and halitosis are associated with impaired oral health-related quality of life (OHRQoL). However, no evidence has been found to clearly associate these oral complaints with saliva secretion rates in patients with pSS [6, 13].

Patients with primary Sjogren’s syndrome (pSS), as well as the physicians who treat them, most frequently focus on dry mouth, dry eyes, fatigue, and joint and muscle pain as relevant signs and symptoms of pSS. Chemosensory dysfunction, oral disorders, burning sensations in the tongue (BST) and halitosis, which negatively affect oral health-related quality of life (OHRQoL), are commonly not recognized.. Moreover, the patients and some physicians are not aware of the fact that chemosensory dysfunction and oral disorders are directly related to pSS. The data regarding chemosensory dysfunction and oral disorders, burning sensations in the tongue and mouth in patients with pSS are limited. Therefore, it is of utmost importance to assess the impaired olfactory and gustatory functions as soon as oral disorders in patients with pSS occur.

Before being diagnosed with Sjögren’s syndrome, patients with oral symptoms are initially examined by a dentist. It is of great importance that dental professionals immediately recognize the signs and symptoms of xerostomia and suspect that these patients may suffer from pSS. In order to prevent the development of chemosensory dysfunction and oral disorders, it is essential to provide patients with pSS with early and appropriate treatment. Management includes intense oral hygiene, prevention of oral infections and their treatment, the use of artificial saliva, as well as local and systematic stimulation of salivary secretion [14]. Good oral hygiene, daily dental care and regular dental check-ups are of utmost importance. In cases of dysgeusia and burning mouth disorder, tricyclic antidepressants and clonazepam can bring some relief, but these drugs could also cause additional mouth dryness in patients with pSS [15]. Topical anaesthetics like lidocaine gel are indicated for severe dysgeusia [16]. Artificial saliva may provide useful relief from xerostomia [17]. Additionally, strategies that capitalize on non-olfactory components of food flavor (altering food texture, primary taste qualities, temperature, and color) should be implemented to help maintain previous levels of food enjoyment. Foods and beverages that are salty, sweet, or those which can stimulate the trigeminal nerve (e.g. black or red pepper, carbonated drinks) could make the eating experience more pleasant. Enhancing the olfactory component of food flavor can also help patients with olfactory dysfunction to increase their food intake. These compensatory strategies may also expand dietary choices thus maintaining food enjoyment in addition to healthy eating habits.

The aim of this comparative cross-sectional study was to evaluate olfactory and gustatory function, burning sensation in the tongue (BST), halitosis, and OHRQoL in patients with primary Sjögren’s syndrome and to compare these findings with those of age- and gender-matched healthy controls. The study hypothesis is that patients with pSS have impaired chemosensory function which has a negative impact on Oral Health-Related Quality Of Life.

## Methods

### Study participants

This comparative cross-sectional study was performed at the Outpatient Clinic of the Institute of Rheumatology, University of Belgrade, Serbia. The period of recruitment was from the end of 2017 till the beginning of 2019. All fifty-eight patients with primary SS met the American–European Consensus (AEC) classification criteria [18]. These criteria include subjective symptoms of ocular dryness; subjective symptoms of oral dryness; objective measururement of ocular dryness by Schirmer’s test or corneal staining; salivary gland biopsy with focus score > 2; salivary scintigraphy showing reduced salivary flow (1.5 mL in 15 min) and/or diffuse sialectasias and positive autoantibodies against SS-A and/or SS-B. Patients were diagnosed with primary SS if 4 out of 6 criteria were met; either salivary gland pathology symptoms or the presence of autoantibodies against SS-A/SS-B are necessary for the diagnosis. If the patients had already been diagnosed with rheumatoid arthritis (RA), systemic lupus erythematosus (SLE) or scleroderma prior to developing their sicca symptoms, they were diagnosed with secondary SS.

Fifty-five healthy controls of similar age and gender-matched were enrolled in this comparative cross-sectional study. The majority of the healthy volunteers were recruited from the healthy staff of the Institute of Rheumatology. Together with other recruited healthy subjects they completed a health questionnaire. All study participants had given their informed consent according to the Declaration of Helsinki, and the study was approved by the Local Ethics Committee at the Institute of Rheumatology. [Mirjana Šijan Gobeljić], [Vera Milić,] and [Nemanja Damjanov] prepared study material, collected data and did all the analyses.

The patients with pSS were aged 25–77 years and were randomly and continuously recruited into the study from the Outpatient clinic of the Institute of Rheumatology. The exclusion criteria for the patients were inflammatory rheumatic diseases or systemic connective tissue diseases, active infections, malignant diseases, metabolic diseases or any other condition that, in the investigator’s opinion, could make a patient ineligible to participate in the study. The exclusion criteria for the healthy controls were subjective mouth and eye dryness, presence of chronic rheumatic, metabolic or malignant diseases.

### Clinical assessment

Disease characteristics (activity of pSS, extraglandular manifestations), patient’s medical history, chronic diseases, use of medications, and unhealty lifestyle habits (such as smoking) were recorded. Schirmer’s I test and Rose Bengal score were used for evaluation of objective xerophthalmia. Compromised function of the major salivary glands was assessed by salivary scintigraphy using radioactive technetium-99 m (Tc99m) pertechnetate, as easy, reproducible and well tolerated techique. Excretion rate was defined as the difference between maximum and minimum excretion after being stimulated by vitamin C, divided by the maximum counts. The excretion level ≥ 50% was classified as normal or dysfunctional if it was < 50% [19, 20].

Labial salivary gland (LSG) biopsies were performed in patients with pSS. The degrees of lymphocytic infiltration of the salivary gland tissue sections (4mm^2^) were scored from 0 to 4, according to the semiquantitative scoring method of Chisholm and Mason [21]. Grade 0 denoted the absence of inflammatory infiltrate; grade 1 slight presence of infiltrate; grade 2 moderate presence of infiltrate of focus score < 1 (focus score is defined as number of aggregates of ≥50 lymphocytes per 4 mm2 of tissue). Grades 3 and 4, which are defined as pathological findings corresponded to focus scores ≥1.

### Laboratory assessment

Laboratory assessment of study participants included routine laboratory and immunoserological tests. Antinuclear antibodies (ANA; positive if titers > 1:80) were measured by indirect immunofluorescence on the HEp-2 cell line substrate (Organtec Diagnostica, Germany). The serum levels of rheumatoid factor (IgM-RF) were determined by laser nephelometry. Anti-Ro/SS-A and anti-La/SS-B antibodies were detected by enzyme-linked immunosorbent assay (ELISA; Organtec Diagnostica) [19, 20].

### Assessment of chemosensory and oral disorders

The participants were instructed not to eat, drink, or smoke for at least 1 h before their appointment at the Institute of Rheumatology. A detailed personal medical history was recorded. Also, the participants were examined by specialists in rheumatology and dentistry.

The olfactory and gustatory assessments were carried out as described below. Before olfactory testing, the subjects were asked to score their own general subjective smell perception on a visual analogue scale (VAS) from 0 to 10, where self-reported smell was scored as 0 = no smell perception up to 10 = very good smell perception. In cognitive evaluation of olfactory function an identification test with 12 odor pens (Sniffin’Sticks-Screening; BurghartMesstechnik, Wedel, Germany) was used. The pens were positioned under the subject’s nose, approximately 2 cm from either nostril, for a maximum of 4 s. The subjects were instructed to choose from the three possible answers (anosmic/hyposmic – 0 points, or normosmic–1 point) for each of 12 odors on a multiple-choice scoring card [22]. The answers chosen by each individual participant were recorded on a protocol sheet, and the data were scored separately for each of them. A normative classification was used to define anosmic (score: 0–5), hyposmic (score: 6–9), and normosmic (score: 10–12) subjects.

Before gustatory testing, the subjects were asked to score their subjective taste perception on VAS of 0–10, where self-reported taste score 0 meant no taste perception and score 10 meant very good taste perception. A gustatory assessment was performed after the subjects had been given a detailed explanation of the testing procedure. Gustatory function was evaluated using taste strips with four basic taste qualities sweet, sour, salty and bitter [23]. The taste strips (length 8 cm, tip area2 cm^2^; Burghart Messtechnik, Wedel, Germany) were gently rubbed onto the anterior tip of the extended tongue. Different taste qualities were presented in a random manner. A chart with names of the four taste qualities was placed in front of the subjects during testing in order to ask the subjects to identify the taste of the strip. The subjects were allowed to rinse their mouths with water during the gustatory testing. Semi-quantitative evaluation for each of four taste qualities was performed as follows: 0 = loss of ability to taste, 1 = reduced ability to taste and 2 = normal ability to taste. This protocol resulted in a total maximum score of 8, for each subject.

The subjects of this study completed a questionnaire for the assessment of dysgeusia, burning sensation in the tongue (BST), and halitosis [9, 10, 12]. In addition, they described their experience of these conditions using open-ended questions. They also completed oral health-related quality of life (OHRQoL) questionnaire using the 14-item short form of the Oral Health Impact Profile (OHIP-14) [24–26]. Serbian version of OHIP-14 was produced after the questionnaire had undergone back translation, linguistic and cultural validation. It had to be linguistically validated (and psychometrically tested) since no Serbian version had been available. Specifically, the questionnaire was translated from English into Serbian by two independent translators. Pretesting of the resulting questionnaire was conducted on a sample of 10 patients before it was used in this study in order to avoid a possible misinterpretation of any of the questions. The translated version proved to be accurate and well understood by the patients, so no further cultural modification seemed to be necessary.

The total OHIP-14 sum score ranged from 0 to 56, giving an overall indication of the patient’s OHRQoL. A high OHIP-14 score indicated a poor OHRQoL.

Disease activity and the presence of extraglandular manifestations were estimated by the EULAR index of disease activity (ESSDAI, range 0–123) [27, 28]. The subjective evaluation of the dryness intensity, joint pain and fatigue were assessed with the use of EULAR SS Patient Reported Index (ESPRI, range 0–10) [29].

### Statistical analyses

Statistical analysis was conducted using the Statistical Package for the Social Sciences (SPSS) version 16.0. The sample sizing procedure was as follows: we defined our Population Size and set our Confidence Level to be 95%, which corresponds to a Z-score of 1.96. Based on the relevant literature, we used 0.5 for SD value, which was an adequate number for our sample size. After defining these values, we used the Sample size formula: Necessary Sample Size = (Z-score)2 * StdDev*(1-StdDev) / (margin of error)_*2.*_

Normality of the data was assessed by Kolmogorov-Smirnov normality test. Independent samples *t*-test was used to compare normally distributed continuous variables in both patient and control groups. A chi-square test was used to compare dichotomous variables. Odds ratios (ORs) with 95% confidence intervals (CIs) for smell or taste alterations in patients with pSS and healthy controls were determined. *P* values < 0.05 were considered statistically significant.

## Results

### Demographical and clinical characteristics of patients with primary Sjögren’s syndrome and healthy controls

The characteristics of patients with pSS and healthy controls are shown in Table 1. The patients with pSS and the healthy controls had a comparable mean age (*p* = 0.917). The majority of subjects in either group were females (*p* = 0.244). The two groups did not differ significantly in smoking habits (*p* = 0.560). In comparison with patients with pSS none of the healthy controls had subjective experience of ocular dryness (< 0.0001), oral dryness (< 0.0001), dysphagia (< 0.0001), nasal dryness (< 0.0001), dyspareunia (< 0.0001), salivary gland enlargement (< 0.0001) or Reynaud phenomenon (< 0.0001). The mean disease duration in patients with pSS was 7.65 ± 5.85 years, ranging from 1 to 22 years. The majority of patients with pSS (43%) had moderate disease activity as estimated by ESSDAI EULAR index of disease activity. More than half of patients (60%) reported that they were not satisfied with their current health status as assessed by EULAR SS Patient Reported Index ESSPRI. The vast majority of patients (more than 88%) had positive salivary gland scintigraphy, salivary gland biopsy and abnormal tear breaking-up time (BUT), Schirmer’s test and Rose Bengal score. The majority of patients (71%) were positive for antinuclear antibodies, SS-A autoantibodies (81%) and RF (69%). The mean value for erythrocyte sedimentation rate (ESR) was 25.7 mm/hour and it ranged from 2 to 88 mm/hour. Leucopenia was found in more than half of the patients (62%). Twenty–two patients with pSS (38%) were on corticosteroid therapy, whereas the majority among them (75%) took 10-50 mg of corticosteroids per day. The majority of patients (81%) used artificial tears, while only 4% of patients used artificial saliva, 46% received Chloroquine or Hydroxychloroquine, 3% Azathioprine and 2% Methotrexate. The majority of patients in this study received drugs that are not expected to have significant effect on chemosensory function.

**Table 1:** Characteristics of patients with primary Sjögren’s syndrome and healthy controls

Characteristic	Patients with pSS (***n*** = 58)	Healthy controls (***n*** = 55)	95% CI of the difference	P
***Age (yrs)*** (mean ± SD)	54.91 ± 13.68	51.42 ± 13.82	−8.62 to 1.63	0.917
***Gender***				
Female	55 (94.8)	55 (100.0)		0.244
***Smoking status***				
Current smoker	13 (22.4)	8 (14.5)		
Former smoker	5 (8.6)	5 (9.1)		0.560
Never smoked	40 (60.9)	42 (76.4)		
***Disease duration (yrs)*** (mean ± SD)	7.65 ± 5.85	N/A	6.09 to 9.22	
***Ocular dryness***	51 (87.9)	0 (0.0)		< 0.0001
***Oral dryness***	53 (91.4)	0 (0.0)		< 0.0001
***Dysphagia***	35 (60.3)	0 (0.0)		< 0.0001
***Nasal dryness***	18 (31.0)	0 (0.0)		< 0.0001
***Dyspareunia***	21 (36.2)	0 (0.0)		< 0.0001
***Salivary gland enlargement***	28 (48.3)	0 (0.0)		< 0.0001
***Reynaud phenomenon***	24 (42.1)	0 (0.0)		< 0.0001
ESSDAI*		N/A		
< 5 low activity	15 (26.8)			
5–13 moderate activity	24 (42.8)			
> 13 high activity	17 (30.4)			
ESSPRI**		N/A		
< 5 satisfactory	23 (40.4)			
> =5 unsatisfactory symptom state	34 (59.6)			
***Scintigraphy of salivary glands***		N/A		
Positive	41 (89.1)			
***Salivary gland biopsy***		N/A		
Positive	28 (87.5)			
***Schirmer’s test***		N/A		
Abnormal	45 (90.0)			
***Rose Bengal score***		N/A		
A bnormal	38 (95.0)			
***Tear break-up time (BUT)***		N/A		
Abnormal	43 (93.5)			
***Antinuclear antibody***		N/A		
Positive	41 (70.7)			
***SS-A***		N/A		
Positive	47 (81.0)			
***SS-B***		N/A		
Positive	27 (46.6)			
***RF***		N/A		
Positive	40 (69.0)			

Values are given as *n* (%); Statistical analysis was performed using chi-square tests except for age (independent samples *t*-test); *(*n* = 57); ** (*n* = 56)

### Olfactory function

**Fig. 1:**
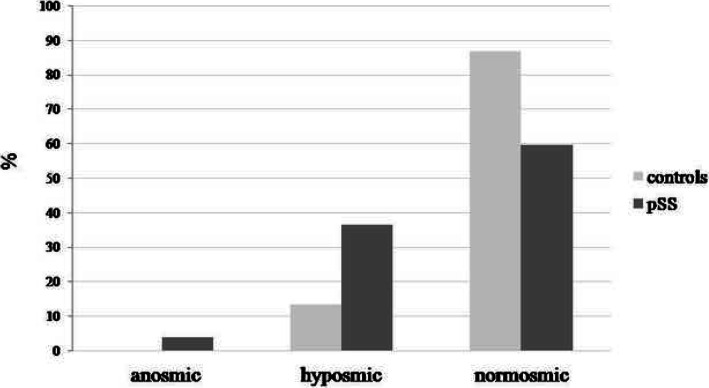
Olfactory function in patients with pSS and healthy controls. Significantly higher frequencies of pSS patients with anosmia and hyposmia in comparison with healthy controls (χ^2^ = 9.9; *p* = 0.002)

The pSS group had a significantly lower mean self-reported smell score on VAS than healthy controls (8.57 ± 2.21 95% CI 7.99 to 9.15 vs. 9.56 ± 0.72 95% CI 9.36 to 9.76; *p* = 0.016; (Table 2). Similarly, olfactory testing showed that the patients with pSS were significantly more anosmic and hyposmic and significantly fewer of them were normosmic in comparison with healthy controls (χ^2^ = 9.9; *p* = 0.002), as shown in Fig. 1.

**Table 2:** Gustatory function in patients with primary Sjögren’s syndrome and healthy controls

Variable	Patients with pSS (*n* = 58)	95% CI	Healthy controls (*n* = 55)	95% CI	P
***Self-reported smell score (VAS)*** (mean ± SD)	8.57 ± 2.21	7.99 to 9.15	9.56 ± 0.72	9.36 to 9.76	0.016
***Self-reported taste score (VAS)*** (mean ± SD)	8.48 ± 2.10	7.93 to 9.04	9.54 ± 0.67	9.35 to 9.73	0.014
***Taste score*** (mean ± SD)	4.11 ± 1.82	3.57 to 6.64	6.11 ± 1.93	5.58 to 6.64	< 0.0001
***Gustatory function***					
*Ability to taste sweet*					
Loss of taste	16 (34.0)		4 (7.5)		
Reduced taste	29 (61.7)		17 (32.1)		< 0.0001
Normal taste	2 (4.3)		32 (60.4)		
*Ability to taste sour*					
Loss of taste	5 (10.6)		0 (0)		
Reduced taste	22 (46.8)		24 (45.3)		0.054
Normal taste	20 (42.6)		29 (54.7)		
*Ability to taste salty*					
Loss of taste	5 (10.0)		3 (5.7)		
Reduced taste	30 (60.0)		21 (39.6)		0.018
Normal taste	15 (30.0)		29 (54.7)		
*Ability to taste bitter*					
Loss of taste	9 (19.1)		1 (1.9)		
Reduced taste	28 (59.6)		27 (50.9)		0.001
Normal taste	10 (21.3)		25 (47.2)		

Values are given as *n* (%); Statistical analysis was performed using Mann-Whitney test (self-reported taste score-VAS, taste score) and chi-square test (gustatory function)

### Gustatory function

The patients with pSS had a significantly lower mean self-reported taste score on VAS than healthy controls (8.48 ± 2.10 95% CI 7.93 to 9.04 vs. 9.54 ± 0.67 95% CI 9.35 to 9.73; *p* = 0.014). Gustatory testing showed that the pSS patient group had significantly lower mean taste scores than the control group (4.11 ± 1.82 95% CI 3.57 to 6.64 vs. 6.11 ± 1.93 95% CI 5.58 to 6.64; *p* < 0.0001) (Table 2). Gustatory testing categorized significantly more patients with pSS as ageusic/hypogeusic and significantly fewer with normal sense of taste than in the control group as shown in Table 2. Significantly more patients with pSS had impaired ability to taste sweet (p < 0.0001), sour (*p* = 0.054), salty (*p* = 0.018) and bitter (*p* = 0.001) than healthy controls.

### Dysgeusia, burning sensation in the tongue, and halitosis

Complaints of dysgeusia, BST, and halitosis in the patients with pSS and healthy controls are shown in Table 3. Only five out of 53 healthy controls complained of dysgeusia, while more than half of patients with pSS (53%) reported dysgeusia (χ^2^ = 23.6, *p* < 0.0001). Thirty patients with pSS who complained of dysgeusia described the taste as metallic, sour, bitter, rotten or unpleasant. The majority of patients reported distorted bitter taste (36.7%), while all controls that reported dysgeusia (9.4%) complained of unpleasant taste. Similar proportions of the patients with pSS (77%) and healthy controls (67%) experienced distorted taste as a daily problem.

**Table 3:** Frequency of dysgeusia, burning sensations in the tongue (BST) and halitosis and Odds ratio for the development of dysgeusia, halitosis and BST in patients with primary Sjögren’s syndrome and in healthy controls

Variable	Patients with pSS (*n =* 57)	Healthy Controls (*n* = 53)	P	Odds ratio	95%CI	P
***Dysgeusia***						
Yes	30 (52.6)	5 (9.4)				
No	27 (47.4)	48 (90.6)	< 0.0001	10.7	3.7–30.7	< 0.001
***Distorted taste***						
Metallic	3 (10.0)	0 (0.0)				
Rotten	3 (10.0)	0 (0.0)				
Bitter	11 (36.7)	0 (0.0)				
Sour	5 (16.7)	0 (0.0)				
Unpleasant	8 (26.7)	5 (100.0)	n.s.			
***Frequency of distorted taste***						
Constantly	2 (6.7)	1 (33.3)				
Daily	23 (76.7)	2 (66.7)	n.s.			
Sometimes	1 (3.3)	0 (0.0)				
In bad periods	4 (13.3)	0 (0.0)				
***BST***						
Yes	26 (45.6)	0 (0.0)				
No	31 (54.4)	53 (100.0)	< 0.0001	^a^-	–	< 0.001
***Frequency of BST***						
Constantly	5 (19.2)	N/A				
Sometimes	5 (19.2)	N/A				
During the meal	10 (38.5)	N/A				
Between the meals	6 (23.1)	N/A				
***Type of BST***						
Harsh	5 (27.8)	N/A				
Sour	7 (38.9)	N/A				
Sweet/Sour	3 (16.7)	N/A				
Other	3 (16.7)	N/A				
***Halitosis***						
Yes	18 (31.6)	15 (28.3)				
No	39 (68.4)	38 (71.7)	0.434	1.2	0.5–2.6	0.708
***Frequency of halitosis***						
Constantly	5 (27.8)	3 (20.0)				
Daily	9 (50.0)	12 (80.0)				
Sometimes	2 (11.1)	0 (0.0)	0.328			
In bad periods	2 (11.1)	0 (0.0)				

Values are given as *n* (%); Statistical analysis was performed using chi-square test; n.s. (not significant)

^a^Odds ratio could not be calculated due to the number < 10 of observations in one group

While none of the controls complained of burning sensation of the tongue (BST), nearly half of patients with pSS reported BST (46%) (χ^2^ = 31.6, *p* < 0.0001). The majority of patients with pSS (38%) experienced burning sensation in the tongue during the meals and 39% of them reported sour taste sensation as a type of BST.

About 32% of patients and 28% of controls complained of halitosis, but the difference between the two groups was not significant (χ^2^ = 0.40, *p* = 0.434). Half of the pSS patients experiencing halitosis complained of halitosis as a persisting daily problem, similarly to the majority of healthy controls (80%) who also reported halitosis as a daily problem (*p* = 0.328).

**Fig. 2:**
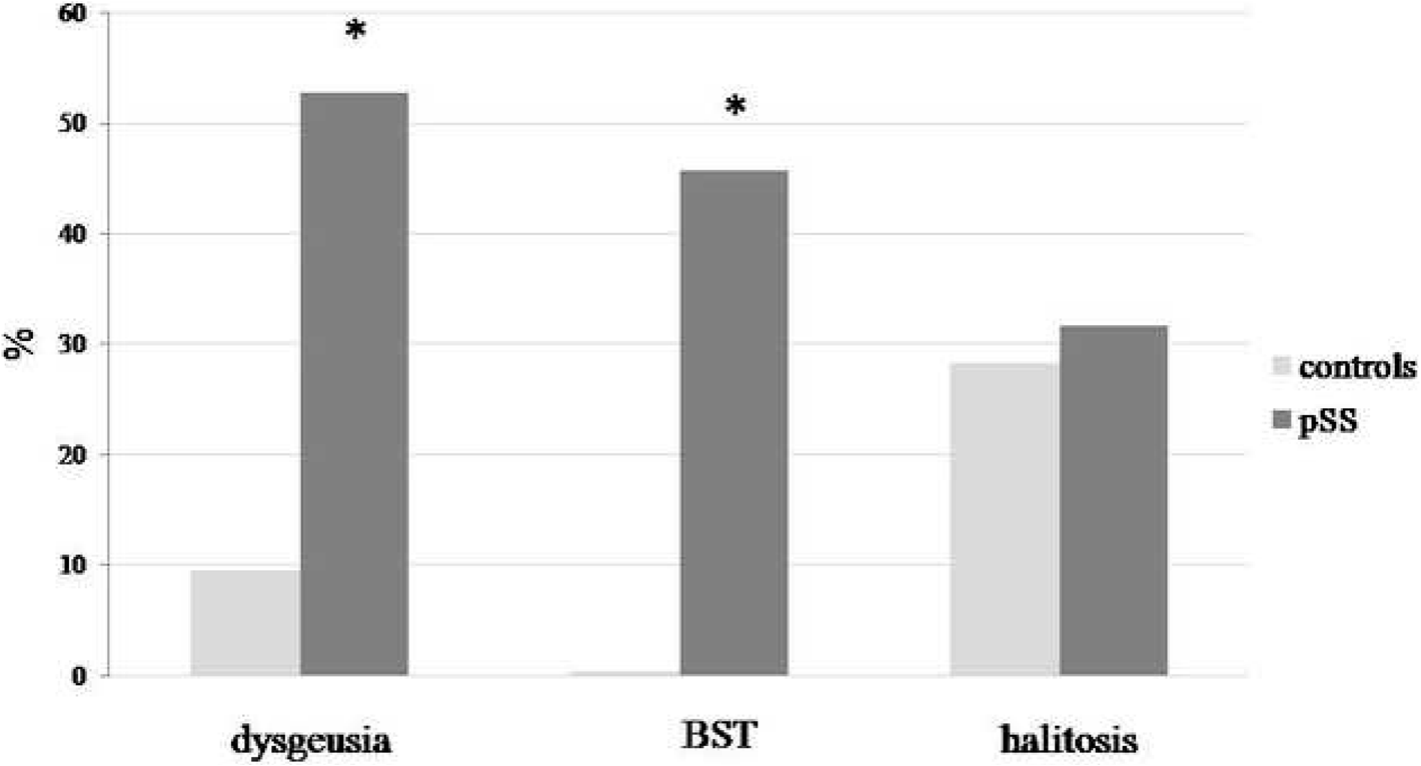
Dysgeusia, burning sensations in the tongue (BST), and halitosis in patients with primary Sjögren’s syndrome and in healthy controls. Significantly higher frequencies of pSS patients with self-reported complaints of dysgeusia (χ^2^ = 23.6, p < 0.0001), BST (χ^2^ = 31.6, p < 0.0001), but not of halitosis (χ^2^ = 0.40, *p* = 0.434) in comparison with healthy controls

Highly significant differences in frequency of self-reported complaints of dysgeusia among patients with pSS and controls and BST were observed, with no differencies in the presence of halitosis as shown in Fig. 2.

Odds ratios for the development of dysgeusia, BST and halitosis were determined in patients with SS and healthy controls and the results are given in Table 3. In addition, positive findings of anosmia (40.4%) were significantly higher among patients with primary Sjögren’s syndrome than among healthy controls (13.2%) (Odds ratio: 5.2, 95% CI: 1.9–14.3, *p* < 0.001). The obtained results show that pSS is a risk factor for the development of dysgeusia, BST and anosmia.

**Fig. 3:**
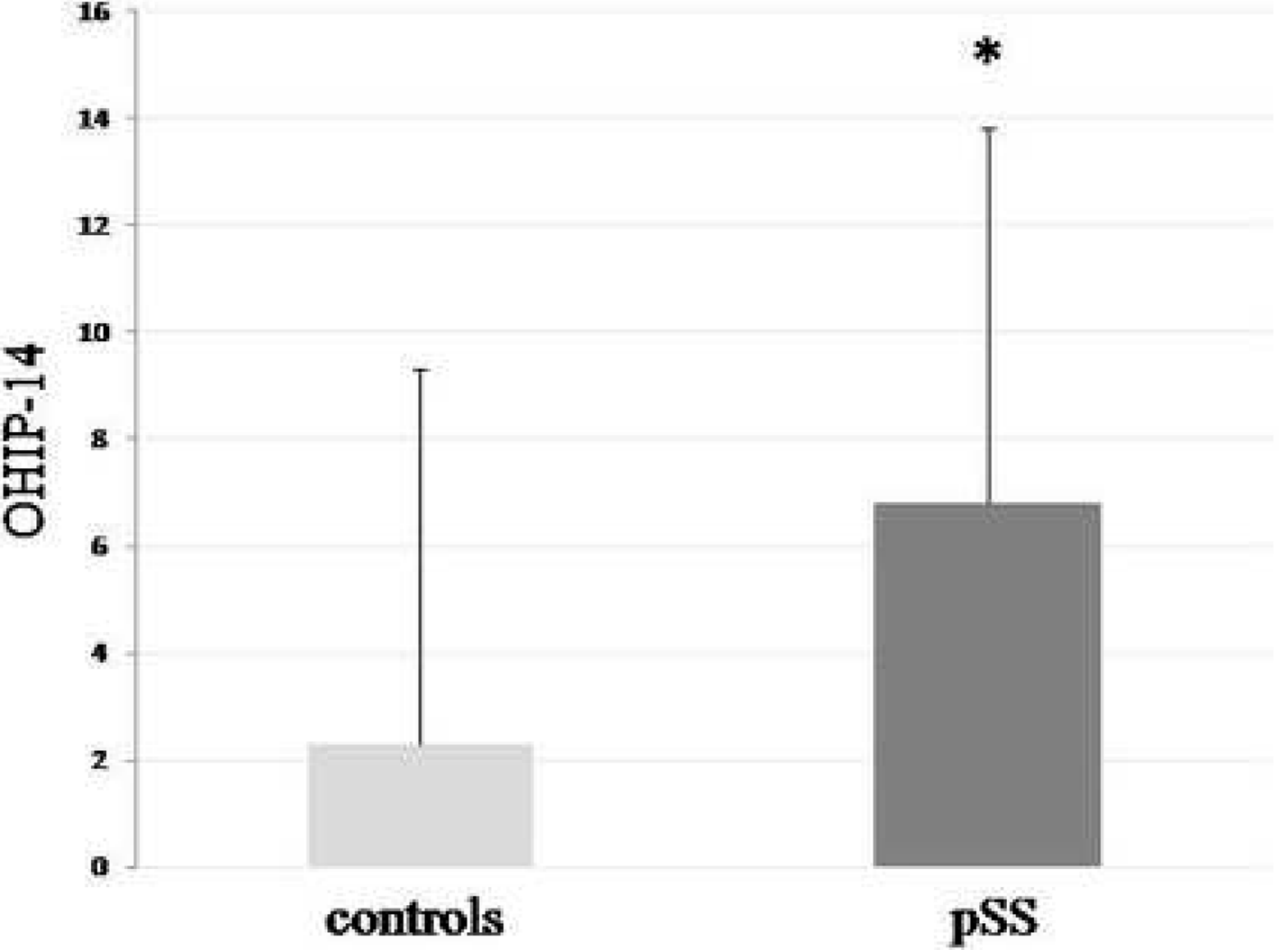
Oral health-related quality of life (OHRQoL) in patients with pSS and healthy controls. Patients with pSS had a significantly higher mean OHIP-14 sum score based on the results of a short-form of Oral Health Impact Profile (OHIP-14) questionnaire than healthy controls reflecting poorer OHRQoL (6.79 ± 7.03; 95% CI −0.19 to 4.73 vs. 2.27 ± 8.46; 95% CI 4.90 to 8.67, *p* < 0.0001; Mann-Whitney U test)

The pSS group had a significantly higher mean OHIP-14 sum score than the control group (6.79 ± 7.03; 95% CI − 0.19 to 4.73 vs. 2.27 ± 8.46; 95% CI 4.90 to 8.67, p < 0.001) (Fig. 3). Scores in all domains of OHIP-14 (functional limitation, physical limitation, psychological limitation, and social limitation) were higher in pSS patients than in controls. The pSS group had a significantly lower mean VASEQ5D sum score than the control group (6.67 ± 2.02 95% CI 6.13 to 7.22 vs. 8.28 ± 1.02 95% CI 7.99 to 8.57; *p* < 0.0001).

## Discussion

The reported data about the associations between chemosensory disturbances, BST, halitosis and OHRQoL in patients with SS are limited. The present study demonstrates that patients with pSS have impaired olfactory and gustatory functions, burning sensation of the tongue (BST) and poor OHRQoL in comparison with the healthy controls without sicca symptoms. We found similar frequencies of halitosis among patients with pSS and the healthy controls. Our findings are in agreement with other studies showing disturbed taste and smell functions in patients with SS [5, 6, 30, 31].

In our study, gustatory dysfunction was more frequently found in patients with pSS than olfactory dysfunction. This finding is consistent with some studies [5, 6, 31], but contradictory to one report [30]. A possible explanation for this discrepancy may be related to the different methods for testing smell function. In our study, detection of cognitive smell function was performed by a smell identification test, whereas in the study by Kamel et al. [30] the chemosensory threshold (which reflects peripheral sensory impairment) was assessed. An ideal testing method for smell function would include threshold assessment, detection and identification tests. However, only identification test was performed in our study. Our findings demonstrate that dysgeusia, BST and halitosis were often reported among patients with pSS, which is in line with the reported data [6]. In contrast, no differences were found in the occurrence of halitosis between pSS and the control group in our study.

There are indications that smell and taste impairments, as well as the burning sensation in the mouth may be caused by hyposalivation [5, 13, 32]. However, some studies show that salivary factors are not responsible for impaired taste performance [32]. A recent study showed lower rates of salivary secretion in patients with pSS, but only a weak correlation was found between salivary secretion rates and the presence of oral disorders. This observation implies that oral disorders are not caused by low salivary flow [6].

We observed relatively high percentage of ageusic and hypogeusic patients within the group of patients with pSS. While ageusia is a rare condition that accounts for less than 1% of patients with chemosensory dysfunction [33–35], the patients with pSS in this study were classified as ageusic as they experienced the inability to taste basic tastes: sweetness (34%), sourness (11%), saltiness (10%) or bitterness (19%). Interestingly, between 40 and 50% of healthy controls were found to be hypogeusic. However, the number of patients with pSS with ageusia/hypogeusia was significantly higher compared to healthy controls.

As for olfactory function, anosmia is the most common complaint of patients with chemosensory disorders [13, 31, 33]. However, in our study 3.8% of the patients with pSS were categorized as anosmic and 36.5% as hyposmic. The proportion of anosmic patients in our study was lower than in the study by Rusthen et al. [6], in which 12.9% of patients with pSS were classified as anosmic. The main reasons why we chose to use Sniffin’ Sticks was because they had already been used in clinical practice and available data from the literature [6]. We applied general hygienic measures to prevent any contamination and we strictly followed the regular recommended procedure of positioning the pens under the subject’s nose, approximately 2 cm from either nostril, for a maximum of 4 s. However, having in mind that the most widely used smell test in the world the University of Pennsylvania Smell Identification Test (UPSIT) has become a ‘gold standard’ in olfactory testing, we shall consider its use in our future research. Approximately half of the patients with anosmia and hyposmia experience changes in food preferences resulting in higher consumption of sugar and seasonings [33–35]. Moreover, their loss or reduced ability to taste affect their eating habits [36]. The patients with chemosensory disorders could either increase or reduce food intake, which may result in an increase or a decrease in their body mass [35].

About half of the patients with pSS complained of BST, while none of healthy controls experienced BST. BST in patients with pSS was mainly related to food intake. Burning sensation in the mouth is frequently found in patients with SS [9]. More than half of the patients with pSS had dysgeusia with distorted taste of bitterness occurring on daily basis, while dysgeusia was reported by less than 10% of healthy controls. The occurrence of dysgeusia and burning sensations in the tongue and mouth in patients with pSS is underestimated and the data about the frequency and severity of these disorders are scarce. In our study we found higher proportion of ageusic and hypogeusic patients with pSS, in comparison to the data from other studies [33–35]. The patients with pSS in our study were categorized as ageusic if they had experienced loss of sense of basic tastes: sweetness (34%), sourness (11%), saltiness (10%) or bitterness (19%). Disagreement with the results of other studies suggests that taste impairments in patients with pSS need to be more thoroughly addressed in future studies. Oral disorders occurred on a daily basis in a large proportion of patients with pSS in our study. This finding highlights the need for more attention to chemosensory disorders in patients with this disease.

Possible underlying cause of high occurrence of olfactory and gustatory dysfunctions in patients with pSS could be systemic inflammatory response such as overexpression of interferon–inducible genes [37]. Toll-like receptor pathways and interferon pathways mediate the inflammatory responses in taste tissue in pSS and may interfere with normal taste transduction and taste-bud cell turnover [38].

Oral malodor has been receiving increasing attention over the last decade. In the present study, a third of the patients with pSS complained of halitosis, whereas similar proportion of healthy controls reported oral malodor. The main oral causes of this disorder and effective treatment strategies are known [39]. However, it has been reported that a third of the patients seeking treatment for halitosis do not actually have oral malodor caused by the production of volatile sulphur compounds, therefore they cannot be categorized as ‘genuine halitosis’ patients [12]. This could explain rather high proportion of healthy controls complaining of halitosis in our study.

Chemosensory and oral disorders, burning mouth syndrome in particular, usually reduce the patients’ quality of life, and ‘psychological dysfunction’ is common in patients with this diagnosis [40]. Consistent with this finding, the present study shows poor OHRQoL as estimated by OHIP-14 score in patients with pSS. The OHIP-14 questionnaire is designed to examine only certain aspects of OHRQoL, so an improved questionnaire is needed to obtain a more accurate evaluation of chemosensory disorders and OHRQoL in patients with pSS [6].

A recently published meta-analysis by Al-Ezzi et al. [41] supplies data from five studies which included 378 patients with pSS. This study provides an assessment of the impact of dryness caused by primary SS on smell, taste and sexual function in female patients, and influence on their quality of life. After a systematic literature review, the authors concluded that pSS has a negative impact on smell, taste, sexual function and quality of life in female patients with pSS.

A most recent study demonstrated significantly high occurrence of dysgeusia, burning mouth sensation, halitosis and reduced taste sensation in non-SS sicca patients and patients with pSS. Although non-SS sicca patients do not fulfill Sjögren’s syndrome classification criteria, they had similar or even worse oral complaints than the patients with pSS [42]. On the whole, having a second comparison group with patients suffering from dry mouth syndrome that was not secondary to an autoimmune disease (diabetes, menopause, hypothyroidism, radiotherapy, age, etc.) would be most valuable in determining whether the differences are due to an autoimmune process or the damage caused by dry mouth syndrome. The weakness of our study is that we did not have this second comparison group. In our future research a group of non-SS sicca patients will be included in order to gain a better insight into the association between some other autoimmune diseases, chemosensory dysfunction and oral disorders. A potential limitation of our study is our sample size and the fact that we have not used chemosensory threshold for the ascertainment of smell function. We believe our control patients to be representative of the general population. We also regard the patients with pSS recruited in this study as representative subjects typical of SS population.

## Conclusion

The results obtained in this study show that the patients with pSS had impaired olfactory and gustatory function. Impaired chemosensory function had negative impact on quality of life in patients with pSS. The occurrence of oral disorders, such as dysgeusia, BST, and halitosis was frequently found in patients with pSS and was associated with poorer OHRQoL compared with age- and gender-matched healthy subjects. Therefore, a regular assessment of chemosensory functions and oral disorders in patients with pSS should be performed. Symptomatic treatment of xerostomia and prevention of infections could improve the oral health-related quality of life in these patients. Future studies of habits related to oral hygiene and dietary intake are needed to ensure improved treatment of oral health problems in patients with pSS.

## Data Availability

The datasets used and/or analyzed during the current study are available from the corresponding author upon reasonable request.

## Abbreviations

pSS: Primary Sjögren’s syndrome
BST: Burning sensations in the tongue
OHRQoL: Oral health-related quality of life
OHIP-14: Oral Health Impact Profile
VAS: Visual analogue scale
SS: Sjögren’s syndrome
sSS: Secondary Sjögren syndrome
DCs: Dendritic cells
M3R: Muscarinic acetylcholine type 3 receptors
SNPs: Single nucleotide polymorphisms
MMP9: Matrix metalloproteinase 9
HLA: Human leukocyte antigen
AECG: American-European consensus group
RA: Rheumatoid arthritis
BST: Burning sensation in the tongue
AEC: American–European Consensus
LSG: Labial salivary gland
ANA: Anti-nuclear antibodies
ELISA: Enzyme-linked immunosorbent assay
RF: Rheumatoid factor
ESSDAI: EULAR index of disease activity
ESPRI: EULAR SS Patient Reported Index
SPSS: Statistical Package for the Social Sciences
ORs: Odds ratios
CIs: Confidence intervals
BUT: Tear breaking-up time
ESR: Erythrocyte sedimentation rate
